# The Contribution of Internal and External Factors to Human Spatial Navigation

**DOI:** 10.3390/brainsci14060585

**Published:** 2024-06-07

**Authors:** Laura Piccardi, Raffaella Nori, Jose Manuel Cimadevilla, María Kozhevnikov

**Affiliations:** 1 Department of Psychology, Sapienza University of Rome, 00185 Rome, Italy; 2 San Raffaele Cassino Hospital, 03043 Cassino, Italy; 3 Department of Psychology, University of Bologna, 40127 Bologna, Italy; raffaella.nori@unibo.it; 4 Faculty of Psychology and Health Research Center, University of Almeria, 04120 Almería, Spain; jcimadev@ual.es; 5 Department of Psychology, National University of Singapore, Singapore 17570, Singapore; 6 Martinos Canter for Biomedical Imaging, Harvard Medical School and Massachusetts General Hospital, Boston, MA 02129, USA

Spatial navigation is a multifaceted cognitive function essential for planning and finding routes in one’s environment [1]. It encompasses the awareness of one’s current position, orienting oneself in space, the identification of the goal location, and the formulation of a navigational path linking these points. 

Various cognitive processes, including memory, attention, spatial updating, mental planning, and problem-solving skills, are intricately involved in navigation [2,3,4]. Additionally, numerous internal and external factors such as age, gender, familiarity with the environment, landmark attributes, and surrounding complexity can influence spatial navigation [5]. With the growing utilization of spatial orientation and navigation assessments in neuropsychological evaluations, neuroscientists are increasingly committed to elucidating the factors that underlie performance in large-scale real spaces.

The primary objective of this Special Issue, titled “The Contribution of Internal and External Factors to Human Spatial Navigation”, was to investigate the roles of various internal and external variables in navigation. Consequently, seven papers authored by distinguished scientists in the field were compiled to address this issue from diverse perspectives.

Markostamou, Morrissey, and Hornberger [6] proposed the significance of internal verbal and imagery-based strategies in spatial memory tasks. The authors demonstrated the synergistic operation of verbal and imagery strategies, which collectively support efficient memory performance. Consequently, a higher utilization of verbal and imagery-based strategies correlated with enhanced retrieval.

Furthermore, bilingualism has emerged as a compelling research subject due to its growing prevalence. Bilinguals and monolinguals exhibit discernible functional and structural brain disparities [7,8,9,10]. Gender dimorphism in spatial learning has been documented in various studies [11,12,13,14,15]; this gender-related performance discrepancy is reversed among bilinguals, with bilingual women outperforming their male counterparts [16]. In this Special Issue, Tyborowska, Wegman, and Janzen [17] addressed the executive control advantages observed in bilinguals and the brain activity associated with spatial navigation [17]. The authors elucidated the differential recruitment of brain networks in bilinguals compared to monolinguals. Bilingual individuals demonstrate increased engagement of executive control and spatial regions, which correlates with superior spatial navigation skills.

The hippocampal system supports spatial memory processes and is subject to modulation by mood disorders such as anxiety and depression. Specific hippocampal regions have been implicated in anxiety-related responses [18,19,20]. Zafar et al. [21] investigated the impact of anxiety and depression traits on spatial navigation. Healthy participants with elevated, though non-clinical, levels of anxiety and depression traits undertook a virtual navigation task. Their performance did not exhibit a positive correlation with mood traits, highlighting the crucial distinction between clinical and non-clinical manifestations of mood disorders.

While previous research suggested that cognitive maps are allocentric and encode visual information about local locations and environmental boundaries in a global coordinate system [22,23], there is evidence for another type of cognitive map, which is egocentric [24,25,26]. These egocentric maps represent spatial information relative to the navigator’s position and orientation, encoding orientation-specific views of landmarks from multiple frames of reference. Kozhevnikov and Puri [27] provided experimental evidence for the existence of these two types of cognitive maps (egocentric and allocentric). They showed that they result from different navigational strategies, namely path integration and allocentric (memory-based)navigation, respectively. Their study is the first to demonstrate that path integration is not just a supplementary process to allocentric navigation but a standalone strategy that, in conjunction with egocentric landmark processing, generates a unique type of egocentric cognitive map.

Moreover, gravity provides essential cues for spatial orientation and the construction of spatial cognitive maps. Vestibular information traverses various nuclei before reaching the retrosplenial and entorhinal cortex, contributing significantly to the formation of intricate spatial representations [28]. Consequently, astronauts have reported difficulties in spatial orientation when subjected to microgravity conditions [29,30,31,32]. To investigate the effects of spaceflight on brain activity, a cohort of astronauts was assessed before and after their missions. Burles and Iaria [33] observed a reduction in neural activity within specific brain regions associated with spatial orientation post-flight. However, no behavioral changes were noted, indicating that participants likely employed complementary cognitive strategies to address spatial challenges under altered gravitational conditions.

Another important external factor contributing to spatial navigation is the salience of a cue [34]. Features like shape, size, proximity to the goal, or luminescence are important factors contributing to salience. In addition, the phenomenon of overshadowing refers to the reduced weight of an individual cue when initially presented in a compound [35,36,37,38]. In the spatial domain, it remains unclear whether overshadowing affects proximal and distal cues similarly, as findings have been inconsistent [39,40]. Deery and Commings [41] employed the virtual Morris water maze paradigm to investigate this issue, revealing that proximal cues exerted greater control over navigation than distal cues, which could be disregarded entirely. Consequently, navigation demands cognitive effort, prompting individuals to streamline their search processes by prioritizing proximal cues.

Finally, gender-based dimorphism has been frequently observed in spatial navigation tasks [11,12,42,43,44,45,46], suggesting that participant gender is another significant factor influencing spatial navigation. In this Special Issue, Tascón et al. [47] elucidated how males and females exhibited divergent spatial orientation performance in a virtual task when the goal location was moved to a new place. Their findings demonstrated the feasibility of challenging spatial abilities through a reversal protocol, even after the task had been accurately acquired.

Collectively, these studies underscore the necessity of considering multiple variables that may contribute to the final outcome when investigating spatial memory. This Special Issue highlights the complex interplay between cognitive processes such as verbal and imagery-based strategies, bilingualism, mood disorders, path integration, and egocentric and allocentric navigation strategies along with internal and external factors like age, gender, familiarity with the environment, attributes of landmarks, and environmental complexity. Continuous research in this field is imperative to comprehensively understand the myriad factors influencing navigation, thereby facilitating the development of novel protocols and approaches. 
